# Variation in Risk-Standardized Mortality of Stroke among Hospitals in Japan

**DOI:** 10.1371/journal.pone.0139216

**Published:** 2015-10-07

**Authors:** Hiroki Matsui, Kiyohide Fushimi, Hideo Yasunaga

**Affiliations:** 1 Department of Clinical Epidemiology and Health Economics, School of Public Health, The University of Tokyo, Tokyo, Japan; 2 Department of Health Policy and Informatics, Tokyo Medical and Dental University Graduate School of Medicine, Tokyo, Japan; Osaka University Graduate School of Medicine, JAPAN

## Abstract

Despite recent advances in care, stroke remains a life-threatening disease. Little is known about current hospital mortality with stroke and how it varies by hospital in a national clinical setting in Japan. Using the Diagnosis Procedure Combination database (a national inpatient database in Japan), we identified patients aged ≥20 years who were admitted to the hospital with a primary diagnosis of stroke within 3 days of stroke onset from April 2012 to March 2013. We constructed a multivariable logistic regression model to predict in-hospital death for each patient with patient-level factors, including age, sex, type of stroke, Japan Coma Scale, and modified Rankin Scale. We defined risk-standardized mortality ratio as the ratio of the actual number of in-hospital deaths to the expected number of such deaths for each hospital. A hospital-level multivariable linear regression was modeled to analyze the association between risk-standardized mortality ratio and hospital-level factors. We performed a patient-level Cox regression analysis to examine the association of in-hospital death with both patient-level and hospital-level factors. Of 176,753 eligible patients from 894 hospitals, overall in-hospital mortality was 10.8%. The risk-standardized mortality ratio for stroke varied widely among the hospitals; the proportions of hospitals with risk-standardized mortality ratio categories of ≤0.50, 0.51–1.00, 1.01–1.50, 1.51–2.00, and >2.00 were 3.9%, 47.9%, 41.4%, 5.2%, and 1.5%, respectively. Academic status, presence of a stroke care unit, higher hospital volume and availability of endovascular therapy had a significantly lower risk-standardized mortality ratio; distance from the patient’s residence to the hospital was not associated with the risk-standardized mortality ratio. Our results suggest that stroke-ready hospitals play an important role in improving stroke mortality in Japan.

## Introduction

Stroke remains a potentially life-threatening disease despite recent advances in stroke management, including intravenous thrombolysis with recombinant tissue plasminogen activator [1] and endovascular therapy (thrombectomy and thrombolysis) for ischemic stroke [2]. Poor prognosis of stroke has been found to be related to various patient backgrounds, such as age, consciousness level on admission [3–6], and modified Rankin Scale on admission [7].

Studies have shown access to and mortality after stroke care to be associated with several hospital-level factors, including hospital volume, type of hospital [8], presence of a stroke care unit [9,10], and distance from hospital to home [11,12]. Regarding hospital volume, reports have indicated mixed results about the volume-outcome relationship for stroke [13–17]. However, hospital mortality of stroke in those studies varied widely owing to the small sample sizes from limited geographic areas. Furthermore, the studies lacked sufficient adjustment for the severity of stroke. Little is known about contemporary hospital mortality in stroke in a nationwide clinical setting and how it varies by hospital in Japan.

Assessing the variation in stroke mortality among hospitals is important in promoting optimal quality of care. In the present study, we used hospital-level risk-standardized mortality ratios (RSMRs) after stroke [18,19] with a national database in Japan. With this approach, we compared stroke mortality among hospitals adjusted for patient-level prognostic factors. We also assessed several hospital-level factors that affect stoke mortality.

## Materials and Methods

### Data Source

The Japanese Diagnosis Procedure Combination database is a national database of in-hospital patients in Japan. Details of the database have been described elsewhere [20]. In brief, the database contains administrative claims and discharge abstract data including the following: unique hospital identifiers; 7-digit postal codes of the patients’ residential areas; patients’ age and sex; diagnoses and comorbidities on admission coded according to the International Classification of Diseases and Related Health Problems, 10th Revision; procedures; Japan Coma Scale (JCS) on admission; modified Rankin Scale (mRS) on admission for stroke patients; days from stroke onset to hospital admission (≤3 days, 4–7 days, or ≥8 days); length of stay; and discharge status. As of 2013, the database included data on approximately 7 million inpatients from more than 1,000 hospitals, representing approximately 50% of all acute-care inpatient hospitalizations in Japan. All the academic hospitals were obliged to participate in the database, but participation of non-academic community hospitals was voluntary.

We also used data from the Survey of Medical Institutions, which is conducted every year by the Ministry of Health, Labour and Welfare in Japan.[21] The Survey of Medical Institutions is a census of hospitals in Japan, including hospital structural information such as the presence of specific physicians in each hospital.

This study was approved by the Institutional Review Board of The University of Tokyo. Because of the anonymous nature of the data, the requirement for informed consent was waived.

### Patient Selection and Data

We included patients aged ≥20 years who were admitted to hospital with a primary diagnosis of stroke within 3 days of stroke onset during the 12 months from April 1, 2012 to March 31, 2013. The International Classification of Diseases and Related Health Problems, 10th Revision, codes I60, I61, and I63 were used to determine the diagnoses of subarachnoid hemorrhage, cerebral hemorrhage, and cerebral infarction, respectively. For a subset analysis, we excluded patients with subarachnoid hemorrhage and focused on those with cerebral hemorrhage or cerebral infarction.

Patient-level data included age, sex, type of stroke (subarachnoid hemorrhage, cerebral hemorrhage, or cerebral infarction), JCS on admission, mRS on admission, length of stay, and in-hospital death. The JCS is widely used in Japan to measure impaired consciousness [22]. A JCS score of 0 indicates alert consciousness. Single-digit scores (1, 2, 3) signify patients who are awake without any stimuli. Double-digit scores (10, 20, 30) denote patients who can be aroused by some stimuli. Triple-digit scores (100, 200, 300) indicate coma. The JCS and Glasgow Coma Scale assessments are well correlated [23]. The mRS on admission consisted of the following: 0 (no symptoms at all); 1 (no significant disability despite symptoms—able to carry out all usual duties and activities); 2 (slight disability—unable to carry out all previous activities, but able to look after own affairs without assistance); 3 (moderate disability—require some help, but able to walk without assistance); 4 (moderately severe disability—unable to walk without assistance and unable to attend to own bodily needs without assistance); and 5 (severe disability—bedridden, incontinent, and requiring constant nursing care and attention) [7].

The hospital-level characteristics included hospital volume, hospital type (academic or non-academic hospital), presence of a stroke care unit, availability of endovascular therapy (thrombectomy and thrombolysis) and presence of neurologists. Hospital volume was defined as the number of patients with stroke treated at an individual facility during the study period. In the Diagnosis Procedure Combination database, we checked the availability of endovascular therapy for each hospital. Data on the presence of neurologists in each hospital was obtained from the Survey of Medical Institutions data.

### Distance from Patient’s Residence to Hospital

Full addresses of the hospitals and postal codes of the patients’ residential areas were converted to latitudes and longitudes with address-match geocoding using ArcGIS software, version 10.3 (Esri Inc., Redlands, CA, USA). The area of the Japanese archipelago is approximately 378 000 km^2^. About two-thirds of which is uninhabited mountainous terrain, and the inhabited area is roughly 121 000 km^2^. There are roughly 140 000 postal codes in Japan (unpublished data). A postal code covers approximately 1 km^2^ on average. The measurements were taken from the center of the postal code to the hospital.

The distance from the patient’s residence to the hospital was calculated using Hubeny’s distance calculation formula [24]. For patient-level analyses, the distance from the patient’s residence to the hospital (in kilometers) was categorized into tertiles. For hospital-level analyses, the median of the distance from the patients’ residences to each hospital was calculated.

### Risk-Standardized Mortality Ratio

First, we constructed a multivariable logistic regression model to predict in-hospital death for each patient with the following patient-level factors: age (as a continuous variable); sex; type of stroke (subarachnoid hemorrhage, cerebral hemorrhage, or cerebral infarction); JCS on admission; and mRS on admission. The predicted probability of death for each patient (ranging from 0 to 1) was calculated using a logistic regression model. We determined the prediction accuracy of the logistic model using the c-index (area under receiver operating curve) [19,25]. A c-index value of 0.5 suggests that the model is no better than random chance in predicting death; a value of 1.0 indicates perfect discrimination.

In this study, RSMR was defined as the ratio of the actual number of in-hospital deaths to the expected number of such deaths for each hospital [18,19]. The expected number of in-hospital deaths was equal to the sum of the predicted probabilities of the patients in each hospital. RSMR was calculated only for facilities with a hospital volume of ≥50.

### Statistical Analyses

We employed chi-square tests to compare crude in-hospital mortality between the categories of each patient-level factor. The averages and standard deviations of RSMR were compared between the categories of each hospital-level factor using *t* tests or analyses of variance. A hospital-level multivariable linear regression was modeled to analyze the association between RSMRs and hospital-level factors (type of hospital, presence of stroke care unit, presence of neurologists, availability of endovascular therapy, hospital volume, and median distance from the patient’s residence to the hospital). Because all hospitals with stroke care unit had neurologists, the variables “stroke care unit” and “presence of neurologists” were merged to a single variable with the following three categories: “absence of neurologists”, “presence of neurologists, without stroke care unit” and “presence of neurologists, with stroke care unit”. Availability of endovascular therapy and hospital volume were merged to a single variable including the following four categories: “hospital volume ≤399, endovascular therapy unavailable”, “hospital volume ≥400, endovascular therapy unavailable”, “hospital volume ≤399, endovascular therapy available” and “hospital volume ≥400, endovascular therapy available”. We also performed a patient-level Cox regression analysis to examine the association of in-hospital death with both patient-level and hospital-level factors. We considered a two-sided p value of less than 0.05 significant. For all the statistical analyses, we employed Statistical Package for Social Sciences version 22.0 (IBM SPSS Corp., Armonk, NY, USA).

## Results

During the study period, we identified 176,753 eligible patients from 894 hospitals. The average (standard deviation) age was 72.8 (13.1) years. Table 1 shows the patient-level characteristics and in-hospital mortality for each category. Overall crude in-hospital mortality was 10.8%. Higher crude in-hospital mortality was significantly associated with the following: higher age; female gender; hemorrhagic stroke; higher JCS; and higher mRS. Non-academic hospitals, hospitals with stroke care units, and higher-volume hospitals (≥600) were more likely to have lower crude in-hospital mortality. Patients with a greater distance from their residence to the hospital were significantly likely to have higher crude in-hospital mortality.

**Table 1:** Number of patients and in-hospital mortality in each category.

	No. of patients	In-hospital death	(%)	*p*
Total	176,753	19,123	(10.8)	
Age (years)				<0.001
≤69	63,627	5,189	(8.2)	
70–79	51,304	4,605	(9.0)	
80–89	49,291	6,765	(13.7)	
≥90	12,531	2,564	(20.5)	
Sex				<0.001
Male	97,981	9,425	(9.6)	
Female	78,772	9,698	(12.3)	
Type of stroke				<0.001
Cerebral infarction	121,783	7,887	(6.5)	
Cerebral hemorrhage	40,789	7,379	(18.1)	
Subarachnoid hemorrhage	14,181	3,857	(27.2)	
Japan Coma Scale on admission				<0.001
0	73,347	1,692	(2.3)	
1	27,608	942	(3.4)	
2	13,904	713	(5.1)	
3	19,540	1,703	(8.7)	
10	11,577	1,283	(11.1)	
20	4,202	673	(16.0)	
30	5,059	1,023	(20.2)	
100	5,441	1,519	(27.9)	
200	8,858	4,338	(49.0)	
300	7,217	5,237	(72.6)	
Modified Rankin Scale on admission				<0.001
0–4	147,996	11,710	(7.9)	
5	20,506	5,514	(26.9)	
Missing data	8,251	1,899	(23.0)	
Type of hospital				<0.001
Non-academic hospitals	155,074	16,569	(10.7)	
Academic hospitals	21,679	2,554	(11.8)	
Stroke care unit				<0.001
No	152,379	16,896	(11.1)	
Yes	24,374	2,227	(9.1)	
Hospital volume per year				<0.001
≤199	49,149	5,592	(11.4)	
200–399	72,768	8,183	(11.2)	
400–599	37,045	3,876	(10.5)	
≥600	17,791	1,517	(8.5)	
Distance from patient’s residence to hospital (km)				<0.001
≤1.8	33,895	3,334	(9.8)	
1.9–3.4	34,120	3,563	(10.4)	
3.5–5.7	34,257	3,699	(10.8)	
5.8–10.7	34,394	3,950	(11.5)	
≥10.8	34,528	3,919	(11.4)	
Missing data	5,559	658	(11.8)	

Table 2 shows the RSMR for each category of hospital-level factors in facilities with a hospital volume of ≥50. The RSMR was significantly lower in academic hospitals, hospitals with a stroke care unit, higher-volume hospitals and hospitals in which endovascular therapy was available. The cut-off value for a median distance from the patient’s residence to the facility was set as 4.3 km so that the number of hospitals were equal between the categories (349 hospitals with median distance ≤4.3 km and 349 hospitals with median distance >4.3 km, excluding 26 hospitals with missing data on median distance). No significant difference in RSMR was evident between hospitals with a median distance from the patient’s residence to the facility of less than 4.3 km and those with a distance greater than that.

**Table 2:** Hospital-level characteristics and RSMR.

	No. of hospitals	RSMR, mean (SD)		*p*
Total	724			
Type of hospital				0.005
Non-academic hospitals	618	1.04	(0.34)	
Academic hospitals	106	0.94	(0.32)	
Presence of neurologists and stroke care unit				
Absence of neurologists	33	1.16	(0.37)	<0.001
Presence of neurologists, without stroke care unit	622	1.03	(0.34)	
Presence of neurologists, with stroke care unit	69	0.89	(0.22)	
Hospital volume per year				0.008
≤199	360	1.06	(0.40)	
200–399	262	1.00	(0.26)	
400–599	78	0.95	(0.24)	
≥600	24	0.90	(0.25)	
Availability of transcatherter thrombolysis				
No	435	1.08	(0.38)	<0.001
Yes	289	0.94	(0.25)	
Hospital volume per year and availability of endovascular therapy				
Hospital volume ≤399, Endovascular therapy unavailable	408	1.08	(0.38)	<0.001
Hospital volume ≥400, Endovascular therapy unavailable	27	1.00	(0.25)	
Hospital volume ≤399, Endovascular therapy available	214	0.95	(0.25)	
Hospital volume ≥400, Endovascular therapy available	75	0.92	(0.24)	
Median distance from patient’s residence to hospital (km)				0.246
≤4.3	349	1.03	(0.38)	
>4.3	349	1.00	(0.28)	
Missing data	26	1.14	(0.38)	

RSMR,; SD, standard deviation

**Fig 1:**
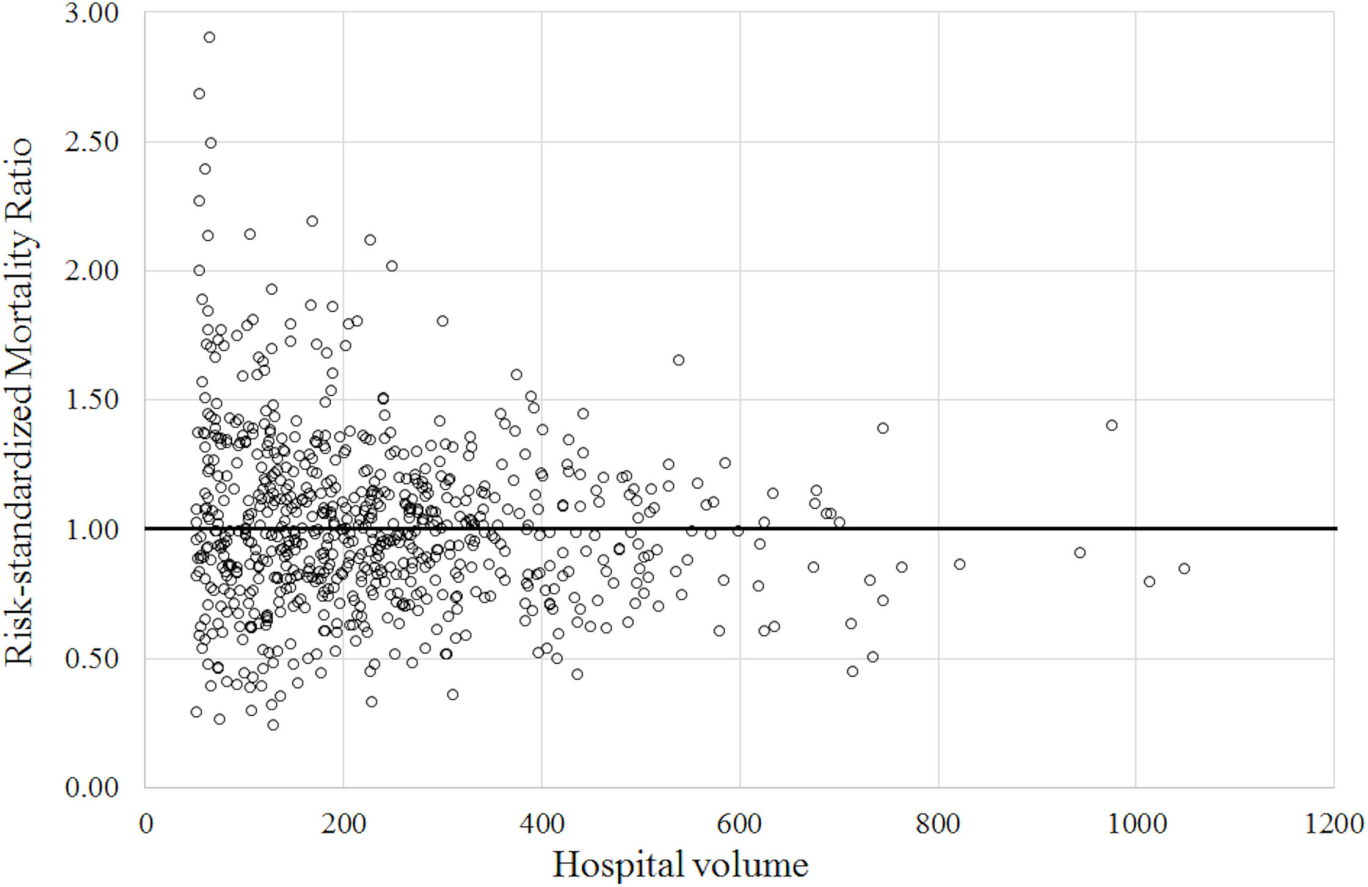
Hospital volume and risk-standardized mortality ratio.

Fig 1 is a scatter diagram of hospital volume and RSMR for the 724 facilities with a hospital volume of ≥50. The numbers of hospitals in the RSMR categories of ≤0.50, 0.51–1.00, 1.01–1.50, 1.51–2.00, and >2.00 were 28 (3.9%), 347 (47.9%), 300 (41.4%), 38 (5.2%), and 11 (1.5%), respectively.

Table 3 shows the results of the hospital-level multivariable linear regression analysis for RSMR. With adjustment for other variables, academic hospitals and hospitals with a stroke care unit were significantly likely to have lower RSMRs. Higher hospital volume and availability of endovascular therapy were significantly associated with lower RSMRs. The median distance from the patient’s residence to hospital was not significantly associated with the RSMR.

**Table 3:** Hospital-level multivariable linear regression for risk standardized mortality ratio (n = 724).

	Coefficient	95% confidence interval			*p*
Type of hospital					
Non-academic hospitals	Reference				
Academic hospitals	-0.07	-0.13	to	0.00	0.058
Presence of neurologists and stroke care unit					
Absence of neurologists	Reference				
Presence of neurologists, without stroke care unit	-0.11	-0.24	to	0.01	0.078
With stroke care unit	-0.20	-0.33	to	-0.06	0.004
Hospital volume per year and availability of endovascular therapy					
Hospital volume ≤399, Endovascular therapy unavailable	Reference				
Hospital volume ≥400, Endovascular therapy unavailable	-0.06	-0.17	to	0.04	0.262
Hospital volume ≤399, Endovascular therapy available	-0.11	-0.16	to	-0.06	<0.001
Hospital volume ≥400, Endovascular therapy available	-0.13	-0.20	to	-0.07	<0.001
Median distance from patient’s residence to hospital (km)					
<4.3	Reference				
≥4.3	-0.02	-0.07	to	0.03	0.437
Missing	0.07	-0.07	to	0.22	0.324
Intercept	1.20	1.08	to	1.33	<0.001

Table 4 shows the results of the Cox regression analysis for in-hospital death (n = 171,194). Patients with higher age, male gender, hemorrhagic stroke, higher JCS on admission, or higher mRS were significantly more likely to die during hospitalization. No significant association was found between in-hospital mortality and distance from the patient’s residence to hospital. After adjustment for these variables, the hazard ratio for the group with a hospital volume of ≥600 was 0.91 (95% confidence interval, 0.86–0.96; *p* = 0.001) compared with the reference group (hospital volume ≤199).

**Table 4:** Cox regression analysis for in-hospital death (n = 171,194).

	Hazard ratio	95% confidence interval			*p*
Age (10-year increase)	1.28	1.26	to	1.29	<0.001
Sex (female)	0.77	0.75	to	0.80	<0.001
Type of stroke					
Cerebral infarction	Reference				
Cerebral hemorrhage	1.52	1.45	to	1.59	<0.001
Subarachnoid hemorrhage	1.43	1.38	to	1.49	<0.001
Japan Coma Scale on admission					
0	Reference				
1	1.25	1.15	to	1.36	<0.001
2	1.63	1.49	to	1.78	<0.001
3	2.60	2.42	to	2.79	<0.001
10	3.13	2.90	to	3.38	<0.001
20	4.34	3.96	to	4.76	<0.001
30	5.35	4.94	to	5.81	<0.001
100	7.58	7.04	to	8.16	<0.001
200	15.47	14.55	to	16.46	<0.001
300	33.69	31.68	to	35.83	<0.001
Modified Rankin Scale on admission					
0–4	Reference				
5	1.21	1.17	to	1.25	<0.001
Type of hospital					
Non-academic hospitals	Reference				
Academic hospitals	0.96	0.92	to	1.00	0.061
Hospital volume per year					
≤199	Reference				
200–399	0.98	0.95	to	1.02	0.39
400–599	0.98	0.94	to	1.02	0.27
≥600	0.91	0.86	to	0.96	0.001
Distance from patient's residence to hospital (km)					
≤ 1.8	Reference				
1.9–3.4	0.99	0.94	to	1.04	0.65
3.5–5.7	0.99	0.94	to	1.04	0.63
5.8–10.7	1.03	0.98	to	1.08	0.25
≥10.8	1.00	0.95	to	1.05	0.98

The results of the subset analysis that focused on those with cerebral hemorrhage or infarction were shown in the supporting tables (S1 Table, S2 Table and S3 Table). These results were almost similar to those in the all-patient analysis.

## Discussion

Using a national inpatient database, this study found that RSMRs varied significantly among hospitals in Japan. Approximately 4% of hospitals had an RSMR of ≤0.50, and approximately 8% of hospitals had an RSMR of >1.50. Academic hospitals, the presence of a stroke care unit, higher hospital volume and the availability of endovascular therapy were associated with lower RSMRs, whereas the distance from the patient’s residence to hospital was not associated with the RSMR.

The present study has several strengths. First, the national inpatient database enabled us to conduct a larger study than previous investigations that assessed the hospital-level factors affecting stroke mortality. Second, this study determined the variation in stroke mortality among hospitals and related factors in a nationwide setting with standardization of patient-level risks.

Previous studies have found that various patient-level prognostic factors affect hospital mortality of stroke. The mRS is a well-known predictor of early mortality for stroke patients [7]. One US study determined that poor prognostic factors for mortality included advanced age, low Glasgow Coma Score, and infratentorial location [4]. In another study, the independent predictors of mortality included male gender, advanced age, hemorrhage location, and decreased consciousness level [5]. The results of the present study are mostly consistent with those of previous studies.

The most notable finding in the present study was a significant disparity in the RSMR of stroke among hospitals. This result can be used as a benchmark for hospitals in seeking better stroke management for improved outcome.

Another important finding of this study was the association between RSMR for stroke and several hospital-level factors. A number of studies have identified a relationship between higher hospital volume and lower stroke mortality [13–17]. However, most reports have lacked sufficient adjustment for patient backgrounds and were based on small sample sizes from limited geographic areas. A recent study using the Diagnostic Procedure Combination database found an association between higher hospital volume and better patient outcomes for ischemic stroke after adjustment for stroke severity [26]. The present investigation determined that variation in RSMR for stroke was partly explained by hospital volume.

In a previous study, academic hospitals were found to have slightly more favorable hospital-level performance in risk-adjusted outcomes [8]. Several reports have shown the presence of a stroke care unit to be associated with a decrease in stroke mortality [9,10]. Our findings confirm superior outcomes in academic hospitals and stroke care units in a nationwide clinical setting.

Our results showed that the availability of endovascular therapy was significantly associated with reduced RSMR. A previous study showed that the majority of patients with acute ischemic stroke did not receive endovascular therapy owing to lack of availability and limited indications for this therapy [26]. Our results suggest that the availability of endovascular therapy is an important part for stroke readiness and for reducing stroke mortality.

Several limitations of the present study should be noted. First, the database only included 50% of acute-care inpatients in Japan. Participating hospitals were skewed to large hospitals because participation of non-academic smaller hospitals was voluntary. Second, although we included a large population, this was a retrospective observational database study in which we could not control the collection or reporting of the variables we were measuring. There could be bias in the form of unmeasured confounders. We were unable to identify several important factors, including National Institute Health Stroke Scale and imaging results (e.g., size of infarction), owing to lack of data availability. Third, the database does not have strict data on discharge to independent living or into care.

In conclusion, this study found that the RSMR for stroke varied widely among hospitals in Japan. Academic status, presence of a stroke care unit, hospital volume and availability of endovascular therapy were significant predictors of lower RSMRs. These results suggest that the availability of stroke-ready hospitals plays an important role in improving mortality.

## Supporting Information

S1 Table Number of patients and in-hospital mortality in each category for those with cerebral infarction or hemorrhage. (DOCX)

S2 Table Hospital-level characteristics and RSMR for cerebral infarction or hemorrhage. (DOCX)

S3 Table Hospital-level multivariable linear regression for risk standardized mortality ratio for cerebral infarction or hemorrhage (n = 724). (DOCX)
