# Supplementary material for: Variation in Risk-Standardized Mortality of Stroke among Hospitals in Japan
Source: PLoS One. 2015 Oct 7;10(10):e0139216. doi: 10.1371/journal.pone.0139216 (PMC4596625; doi:10.1371/journal.pone.0139216)
Supplement: S1 Table — (DOCX) [file pone.0139216.s001.docx]

S1 Table. Number of patients and in-hospital mortality in each category for those with cerebral infarction or hemorrhage

|  | No. of patients | In-hospital death | (%) | *p* |
| --- | --- | --- | --- | --- |
| Total | 162,572 | 15,266 | (9.4) |  |
| Age (years) |  |  |  | <0.001 |
| ≤69 | 55,205 | 3,478 | (6.3) |  |
| 70–79 | 48,162 | 3,704 | (7.7) |  |
| 80–89 | 47,076 | 5,787 | (12.3) |  |
| ≥90 | 12,129 | 2,297 | (18.9) |  |
| Sex |  |  |  | <0.001 |
| Male | 93,337 | 8,107 | (8.7) |  |
| Female | 69,235 | 7,159 | (10.3) |  |
| Type of stroke |  |  |  | <0.001 |
| Cerebral infarction | 121,783 | 7,887 | (6.5) |  |
| Cerebral hemorrhage | 40,789 | 7,379 | (18.1) |  |
| Japan Coma Scale on admission |  |  |  | <0.001 |
| 0 | 70,583 | 1,555 | (2.2) |  |
| 1 | 25,624 | 831 | (3.2) |  |
| 2 | 13,210 | 662 | (5.0) |  |
| 3 | 18,909 | 1,630 | (8.6) |  |
| 10 | 10,053 | 1,165 | (11.6) |  |
| 20 | 3,747 | 614 | (16.4) |  |
| 30 | 4,487 | 941 | (21.0) |  |
| 100 | 4,555 | 1,297 | (28.5) |  |
| 200 | 7,024 | 3,480 | (49.5) |  |
| 300 | 4,380 | 3,091 | (70.6) |  |
| Modified Rankin Scale on admission |  |  |  | <0.001 |
| 0–4 | 137,168 | 9,375 | (6.8) |  |
| 5 | 17,962 | 4,434 | (24.7) |  |
| Missing data | 7,442 | 1,457 | (19.6) |  |
| Type of hospital |  |  |  | 0.246 |
| Non-academic hospitals | 143,709 | 13,451 | (9.4) |  |
| Academic hospitals | 18,863 | 1,815 | (9.6) |  |
| Stroke care unit |  |  |  | <0.001 |
| No | 140,493 | 13,561 | (9.7) |  |
| Yes | 22,079 | 1,705 | (7.7) |  |
| Hospital volume per year |  |  |  | <0.001 |
| ≤199 | 45,825 | 4,580 | (10.0) |  |
| 200–399 | 66,626 | 6,388 | (9.6) |  |
| 400–599 | 33,801 | 3,083 | (9.1) |  |
| ≥600 | 16,320 | 1,215 | (7.4) |  |
| Distance from patient’s residence to hospital (km) |  |  |  | <0.001 |
| ≤1.8 | 31,862 | 2,753 | (8.6) |  |
| 1.9–3.4 | 31,733 | 2,855 | (9.0) |  |
| 3.5–5.7 | 31,505 | 2,929 | (9.3) |  |
| 5.8–10.7 | 31,478 | 3,113 | (9.9) |  |
| ≥10.8 | 30,821 | 3,078 | (10.0) |  |
| Missing data | 5,173 | 538 | (10.4) |  |
