# Supplementary material for: Variation in Risk-Standardized Mortality of Stroke among Hospitals in Japan
Source: PLoS One. 2015 Oct 7;10(10):e0139216. doi: 10.1371/journal.pone.0139216 (PMC4596625; doi:10.1371/journal.pone.0139216)
Supplement: S2 Table — (DOCX) [file pone.0139216.s002.docx]

S2 Table. Hospital-level characteristics and RSMR for cerebral infarction or hemorrhage

|  | No. of hospitals | RSMR, mean (SD) | | *p* |
| --- | --- | --- | --- | --- |
| Total | 724 |  |  |  |
| Type of hospital |  |  |  | 0.030 |
| Non-academic hospitals | 618 | 1.03 | (0.37) |  |
| Academic hospitals | 106 | 0.94 | (0.35) |  |
| Presence of neurologists and stroke care unit |  |  |  | <0.001 |
| Absence of neurologists | 33 | 1.16 | (0.37) |  |
| Presence of neurologists, without stroke care unit | 622 | 1.03 | (0.34) |  |
| Presence of neurologists, with stroke care unit | 69 | 0.89 | (0.22) |  |
| Hospital volume per year |  |  |  | 0.060 |
| ≤199 | 360 | 1.05 | (0.42) |  |
| 200–399 | 262 | 1.00 | (0.30) |  |
| 400–599 | 78 | 0.96 | (0.26) |  |
| ≥600 | 24 | 0.90 | (0.26) |  |
| Availability of transcatherter thrombolysis |  |  |  | <0.001 |
| No | 435 | 1.06 | (0.41) |  |
| Yes | 289 | 0.94 | (0.28) |  |
| Hospital volume per year and  availability of endovascular therapy |  |  |  | <0.001 |
| Hospital volume ≤399,  Endovascular therapy unavailable | 408 | 1.07 | (0.41) |  |
| Hospital volume ≥400  Endovascular therapy unavailable | 27 | 1.00 | (0.28) |  |
| Hospital volume ≤399,  Endovascular therapy available | 214 | 0.95 | (0.29) |  |
| Hospital volume ≥400  Endovascular therapy available | 75 | 0.93 | (0.25) |  |
| Median distance from patient’s residence to hospital (km) |  |  |  | 0.236 |
| ≤4.3 | 349 | 1.02 | (0.41) |  |
| >4.3 | 349 | 1.00 | (0.30) |  |
| Missing data | 26 | 1.13 | (0.42) |  |

RSMR, risk-standardized mortality ratio; SD, standard deviation
