# Supplementary material for: Variation in Risk-Standardized Mortality of Stroke among Hospitals in Japan
Source: PLoS One. 2015 Oct 7;10(10):e0139216. doi: 10.1371/journal.pone.0139216 (PMC4596625; doi:10.1371/journal.pone.0139216)
Supplement: S3 Table — (DOCX) [file pone.0139216.s003.docx]

S3 Table. Hospital-level multivariable linear regression for risk standardized mortality ratio for cerebral infarction or hemorrhage (n=724)

|  | Coefficient | 95% confidence interval | | | *p* |
| --- | --- | --- | --- | --- | --- |
| Type of hospital |  |  |  |  |  |
| Non-academic hospitals | Reference |  |  |  |  |
| Academic hospitals | -0.05 | -0.13 | to | 0.02 | 0.162 |
| Presence of neurologists and stroke care unit |  |  |  |  |  |
| Absence of neurologists | Reference |  |  |  |  |
| Presence of neurologists, without stroke care unit | -0.11 | -0.25 | to | 0.04 | 0.143 |
| With stroke care unit | -0.20 | -0.35 | to | -0.04 | 0.012 |
| Hospital volume per year and  availability of endovascular therapy |  |  |  |  |  |
| Hospital volume ≤399,  Endovascular therapy unavailable | Reference |  |  |  |  |
| Hospital volume ≥400  Endovascular therapy unavailable | -0.05 | -0.17 | to | 0.07 | 0.400 |
| Hospital volume ≤399,  Endovascular therapy available | -0.09 | -0.15 | to | -0.04 | 0.001 |
| Hospital volume ≥400  Endovascular therapy available | -0.11 | -0.18 | to | -0.04 | 0.001 |
| Median distance from patient’s residence to hospital (km) |  |  |  |  |  |
| <4.3 | Reference |  |  |  |  |
| ≥4.3 | 0.00 | -0.06 | to | 0.05 | 0.958 |
| Missing | 0.08 | -0.08 | to | 0.24 | 0.322 |
| Intercept | 1.17 | 1.03 | to | 1.32 | <0.001 |
